# Elucidation of the effects of caffeine consumption on estrous cycle, hematology, and lipid profile of female Wistar rats

**DOI:** 10.1038/s41598-025-17309-2

**Published:** 2025-10-02

**Authors:** Eunice Ogunwole, Oluwafunmbi Ebenezer Ogunmiluyi, Adedayo Oluwadamilola Adesida, Praise Oluwaseyi Akinwa, Atinuke Oseyemi Akinya, Oluwaseun Alexander Fakeye, Beloved Oyetola Oyetoyan

**Affiliations:** 1 Department of Physiology, University of Medical Sciences, Ondo City, Nigeria; 2 Department of Biosciences and Biotechnology, University of Medical Sciences, Ondo City, Nigeria

**Keywords:** Caffeine, Lipid profile, Hematology, Estrous cycle, Rat, Biochemistry, Physiology

## Abstract

The consumption of caffeinated beverages has become deeply ingrained in modern society, with coffee, tea, and energy drinks being among the most commonly consumed sources of caffeine. Its physiological effects on the cardio-metabolic system have been reported in males. Hematological parameters and lipid profiling are crucial in the diagnosis of cardio-metabolic disorders. Given the growing prevalence of cardiovascular diseases among women and the potential role of caffeine consumption in modulating lipid profiles and hematopoiesis, a comprehensive investigation into this relationship was warranted using a female animal model. The phase of recovery from its effects was also considered in this study. Thirty-five adult female Wistar rats were randomly divided into seven groups (*n* = 5). Group I was the control and received distilled water (0.2 mL/Kg/day). Groups II-IV received daily oral doses of caffeine (10, 20, and 40 mg/Kg/day, respectively) for 21 days. Groups V–VII received similar caffeine doses for 21 days, followed by a 21-day withdrawal (recovery) period. The estrous cycle was assessed by unstained and staining techniques, full blood indices was determined by a hematology analyzer. Serum Tumor necrotic factor-alpha (TNF-α), lipid and hormonal profiles were assayed by ELISA techniques. Statistical analysis employed one way ANOVA with significance taken at *p* < 0.05. The findings of this study showed that caffeine reduced the kidney and liver weights, reduced white blood cell count, Gonadotropin releasing hormone level and further irreversibly reduced progesterone levels, but maintained the lipid profile level. Caffeine increased the level of TNF-α, but adversely impacted the phases of the estrous cycle by reducing their occurrences. These alterations were however reversed during withdrawal from caffeine. Hence, this study highlights that though caffeine may impact immune function, cardiovascular, reproductive and metabolic health, the withdrawal or stoppage of its intake can result in restoration of the altered changes in the body overtime.

## Introduction

Caffeine, one of the most widely consumed psychoactive substances globally, is present in various beverages and dietary supplements^1^. In recent decades, the consumption of caffeinated beverages has become deeply ingrained in modern society, with coffee, tea, and energy drinks being among the most commonly consumed sources of caffeine^2,3^. Its stimulant properties are well-documented, primarily attributed to its antagonism of adenosine receptors in the central nervous system^4^. Caffeine modulates the contractile force in skeletal muscles due to its ability to induce calcium release from the sarcoplasmic reticulum and inhibit its reuptake^5,6^. Similarly, epidemiological human studies have investigated the link between caffeine and atrial and ventricular arrhythmias. Even though caffeine has antifibrotic properties^7^, its consumption, especially at high doses, leads to palpitations and arrhythmias^8^, such as atrial fibrillation and supraventricular and premature ventricular contractions^9,10^.

The consumption of excessive amounts of caffeine has raised concerns in several countries because of its various forms and concentrations, especially when it’s a main component of drinks that include sugar-sweetened beverages^11^. Tempel et al.^12^ found that caffeine intoxication is a major factor in the morbidity and mortality linked to chronic and non-communicable diseases, along with obesity, cardiovascular disease, stroke, and certain malignancies.

Caffeine has been implicated in causing an increase in fatty acid oxidation^13,14^. This is the ability of caffeine to switch the substrate preference from glycogen to lipids by inhibiting glycogen phosphorylase activity and concurrently stimulating hormone-sensitive lipase (HSL) activity^15^. HSL activity is an epinephrine-sensitive lipolytic activity in adipose tissue. Lipolysis, defined as the hydrolytic cleavage of ester bonds in triglycerides (TGs), generates fatty acids (FAs) and glycerol. High blood levels of low-density lipoprotein (LDL) cholesterol, triglycerides, or both have been said to increase the risk of developing cardiovascular diseases. Assessment of lipid profile reveals the status of the fat and cholesterol, which are the major damaging factors to the blood vessel, which in turn can lead to cardiovascular diseases^16,17^. However, Braojos et al.^18^ reported that supplementation with coffee and cocoa By-products helps to ameliorate metabolic syndrome alterations induced by high-fat diets in female mice.

Caffeine’s impact on reproductive functions and fertility has become a popular issue. In premenopausal women, there are reported associations between caffeine intake and fluctuations in reproductive hormone levels^19,20^, raising questions about the potential impact of caffeine on fertility. High caffeine intake during pregnancy has been linked to adverse pregnancy outcomes, including increased risk of miscarriage, preterm birth, and low birth weight^21^. The precise mechanisms underlying the effects of caffeine on the female reproductive system remain incompletely understood. Caffeine’s stimulatory effects on the central nervous system may disrupt hormonal signaling pathways involved in menstrual cycle regulation and reproductive function^22^. Evaluating blood flow patterns, hormonal changes, menstrual cycle, inflammation, and gene expression in caffeine-exposed rats can provide clues about the underlying mechanisms behind caffeine’s influence on the reproductive system^23,24^. TNF-alpha is a pro-inflammatory cytokine that plays a pivotal role in orchestrating immune responses and inflammation, produced primarily by activated macrophages and other immune cells. TNF-alpha functions through specific receptors to regulate immune cell activation, apoptosis, and inflammation pathways^25^. The influence of caffeine on TNF-alpha can be complex and context-dependent. Studies have shown that caffeine can inhibit the production of TNF-alpha in certain immune cells.

The hematological system serves as a vital indicator of overall health status, with alterations in blood parameters often reflecting underlying physiological disturbances. High levels of platelets and leukocytes contribute to inflammation and endothelial dysfunction, promoting the development of atherosclerosis, thrombosis, and plaque rupture^26^, thereby increasing the risk of cardiovascular diseases such as heart attack and stroke^27^. While acute caffeine intake has been associated with transient changes in blood pressure and heart rate^28^, the chronic effects of caffeine on hematological parameters such as red blood cell count, white blood cell count, and platelet count remain poorly understood. Investigating these parameters in a female cohort is particularly pertinent given the influence of hormonal fluctuations on hematopoiesis and immune function.

Given the growing prevalence of cardiovascular diseases among women, infertility issues, and the potential role of caffeine consumption in modulating blood and lipid profiles, there is a noticeable dearth of research focusing specifically on its long-term consequences and the possible impacts of recovery from its intake, particularly in females. Hence, this study utilizes the female animal model to elucidate the mechanisms underlying the effects of chronic caffeine consumption, its recovery on the estrous cycle, hematology, and lipid profile. Exploring this recovery phase can reveal how biochemical systems restore balance after caffeine exposure, providing insights into optimal consumption patterns and potential health implications.

## Materials and methods

### Experimental animal

Adult female Wistar rats (170–200 g body weight) were obtained from the MCtemmy animal house in Ogbomoso, Oyo State, Nigeria. All animals were housed in well-ventilated wire mesh cages under laboratory conditions of a 12-hour dark-light period at 24–25 °C and allowed free access to standard rat pellets and clean water. The animals were acclimatized for two weeks before the administration of caffeine. All procedures involving the use of animals conformed with the Animal Research: Reporting of in Vivo Experiments (ARRIVE) guidelines (2010). Upon obtaining approval, ethical standards and guidelines of the University of Medical Sciences Research and Ethics Committee (UNIMED-AREC) were diligently followed throughout the experiment.

### Experimental design

Thirty-five adult female rats were randomly divided into seven groups (*n* = 5). Group I served as the control and received distilled water. Groups II-IV received daily oral doses of caffeine (10, 20, and 40 mg/kg body weight, respectively) for 21 days. Groups V–VII received similar caffeine doses for 21 days, followed by a 21-day withdrawal period. The weights of the animals were taken during the experimental period, once a week, using an electronic digital weighing scale (EK5055, China). The body weight was measured on the day of sacrifice as well.

### Caffeine preparation

Caffeine was obtained from Central Drug House Ltd. Corp. India. It was freshly prepared by dissolving in distilled water for the animals and administered orally at 10, 20, and 40 mg/Kg body weight daily. The dosage regime was based on previous human and experimental rat studies^29–31^.

### Vaginal smear extraction technique and determination of estrous cycle pattern

The estrous cycle was determined by day-to-day microscopic interpretation of the vagina smear at a specific time (between 7:00 am and 8:00 am) in the morning which was maintained throughout the experiment. The rats were held in place with one hand around the waist with the ventral surface downward for support, while the other hand was used to keep the pipette. The vaginal content was collected with a Pasteur pipette containing about 0.1 mL of normal saline (0.9% NaCl) by gently inserting the tip of the pipette into the rat’s vagina. The introduction was relatively shallow (approximately 1 cm) to avoid excessive cervical stimulation and a consequent pseudo-pregnancy^32^. The pipette was pressed to release the fluid content 2 or 3 times which made a vaginal lavage that contained some of the vaginal cells of the rat. The fluid and its content were thereafter siphoned with a pipette, and the content was placed on a glass slide (A new and clean glass slide was used for each animal). Each glass slide was covered with a cover slip to ensure the smear was in uniform orientation, making it easier to focus and avoid smear coalescing during movement. Some smears were stained using Papanicolaou’s stain and allowed to dry before viewing^33^. The smear was viewed using the ×10 and ×40 magnification objective lenses of the microscope (Olympus, Japan). The cell types and the proportion among them were used to determine the estrous cycle phase of the rat^34–36^.

### Animal blood collection

Following the experimental procedures, the rats were euthanized by cervical dislocation. A midline incision was made along the linea alba, extending from the anterior abdominal wall to the thoracic cavity to expose the heart and internal organs. The weight of each organ was immediately measured using a digital electronic scale (model EHA501, China). Blood was collected via cardiac puncture into plain serum bottles. After allowing the blood to clot for at least 45 min, samples were centrifuged at 3500 rpm for 15 min. The resulting supernatant (serum) was then carefully aspirated and stored at −20 °C for hormonal assay, lipid, and hematological profiling.

### Serum hormonal concentration level by the enzyme-linked immunosorbent assay (ELISA) method

The serum Gonadotropin-Releasing Hormone and Progesterone levels were determined per animal using the ELISA kit (Fortress Diagnostics, UK) according to the protocol in the respective manufacturer’s manual.

### Measurement of TNF-alpha levels

Serum TNF-alpha levels were quantified using an enzyme-linked immunosorbent assay (ELISA) kit specific for rat TNF-alpha. ELISA was performed according to the manufacturer’s instructions.

### Determination of hematological parameters

Centrifuged blood homogenates were placed into the automated hematology analyzer (URIT BT-3000 model), and then the readings of hematological parameters of interest were taken. The hematology analyzer filtered the sample and automatically printed the result.

### Lipid profiling

Serum levels of high-density lipoproteins (HDL), low-density lipoproteins (LDL), cholesterol, and triglyceride were assayed using a colorimetric assay kit (Fortress Diagnostics USA). All procedures were followed as stated in the manufacturer’s instruction guide for the kits.

### Statistical analysis

GraphPad Prism version 8.0 (GraphPad Software, San Diego, USA) was used for the analysis of data obtained from the study. The data were expressed as Mean ± standard error of the mean (SEM) (*n* = 5 per group). The mean differences were compared by a one-way analysis of variance (ANOVA) followed by the Dunnett and Tukey *post hoc test* for multiple comparisons, where necessary, while *p* < 0.05 was considered statistically significant.

## Results

### Effect of caffeine on the percentage change in body weight

**Fig. 1:**
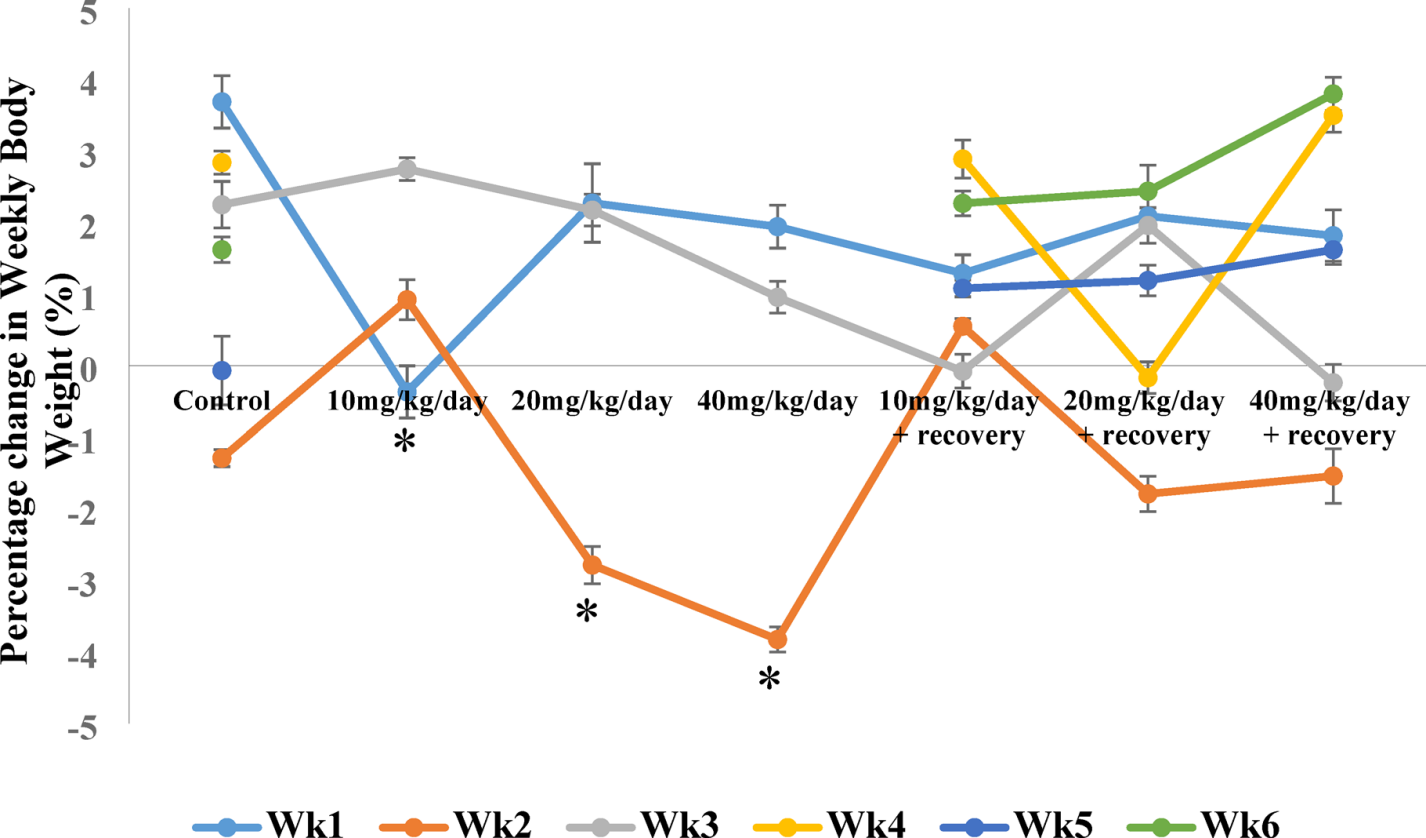
Effect of caffeine on the percentage change in body weight. Bars expressed as Mean ± standard error of the mean (SEM) (*n* = 5 per group). The mean differences were compared by a one-way analysis of variance (ANOVA) followed by the Dunnett *post hoc test* for multiple comparisons. *P* < 0.05. Week 1- ^*^p < 0.05 compared to control.

The results in Fig. 1, show that during the first week, there was a significant (*p* < 0.05) decrease in the percentage change in body weight of the 10 mg/Kg/day caffeine-treated group relative to the control. The weights were also significantly reduced during the second and third week of caffeine intake in the 20 and 40 mg/Kg/day treated groups, which were slightly reversed during caffeine withdrawal as shown in the recovery groups.

### Effect of caffeine on relative organ weight

The kidney and liver weights of all treated groups were significantly decreased compared to the control. The decreased weights were sustained even after caffeine had been withdrawn as revealed by the recovery group (Table 1).

**Table 1:** Effect of caffeine on the relative organ weight.

Group	Control	10 mg/Kg/day	20 mg/Kg/day	40 mg/Kg/day	10 mg/Kg/day + Recovery	20 mg/Kg/day + Recovery	40 mg/Kg/day + Recovery
Kidney	1.40 ± 0.04	1.04 ± 0.06^*^	1.20 ± 0.06^*^	1.20 ± 0.03^*^	0.50 ± 0.03^*^	0.60 ± 0.02^*^	0.70 ± 0.10^*^
Liver	8.00 ± 0.34	6.26 ± 0.1^*^	6.70 ± 0.34^*^	6.70 ± 0.28^*^	6.20 ± 0.24^*^	6.50 ± 0.29^*^	7.80 ± 0.70
Spleen	1.00 ± 0.05	0.69 ± 0.0^*^	0.90 ± 0.07	0.90 ± 0.05	0.70 ± 0.07^*^	0.90 ± 0.09	1.01 ± 0.05

Data expressed as Mean ± standard error of the mean (SEM) (*n* = 5 per group). The mean differences were compared by a one-way analysis of variance (ANOVA) followed by the Dunnett *post hoc test* for multiple comparisons^*^P < 0.05 relative to control.

### Effect of caffeine on hematological parameters

The result of the full blood indices (Table 2) shows a significant (*p* < 0.05) decrease in the white blood cell count of the group treated with 40 mg/Kg/day as compared with 10 mg/Kg/day caffeine treated group. A significant (*p* < 0.05) increase in the Mean corpuscular volume was observed in the group 40 mg/Kg/day + recovery when compared with 10 mg/Kg/day + recovery, also, mean corpuscular hemoglobin concentration was significantly (*p* < 0.05) increased in the 10 mg/Kg/day + Recovery compared with the control, but decreased significantly (*p* < 0.05) in the groups 20 mg/Kg/day + Recovery and 40 mg/Kg/day + Recovery compared with the 10 mg/Kg/day + Recovery group.

**Table 2:** Effect of caffeine on hematological Parameters.

Group	Control	10 mg/Kg/day	20 mg/Kg/day	40 mg/Kg/day	10 mg/Kg/day + Recovery	20 mg/Kg/day + Recovery	40 mg/Kg/day + Recovery
WBC (10^9/L)	9.11 ± 1.59	14.7 ± 2.01	9.14 ± 1.90	6.63 ± 1.70^*^	10.1 ± 2.33	11.7 ± 0.74	13.2 ± 1.48
RBC (10^12/L)	7.11 ± 0.13	6.49 ± 0.17	6.54 ± 0.20	5.87 ± 0.81	5.56 ± 1.19	6.37 ± 0.13	6.56 ± 0.28
HGB (g/dL)	14.0 ± 0.19	13.0 ± 0.46	13.0 ± 0.42	11.5 ± 1.68	11.4 ± 2.37	12.7 ± 0.26	14.0 ± 0.54
HCT (%)	36.4 ± 0.51	33.8 ± 0.92	34.4 ± 1.24	30.4 ± 5.02	27.6 ± 5.95	34.0 ± 0.63	36.6 ± 1.26
MCV (fL)	51.2 ± 0.59	52.1 ± 0.82	52.7 ± 0.60	49.3 ± 2.71	49.4 ± 1.19	53.6 ± 1.71	56.0 ± 1.12^**#**^
MCH (pg)	19.6 ± 0.31	20.0 ± 0.55	19.8 ± 0.13	18.9 ± 0.41	20.7 ± 0.74	20.0 ± 0.67	21.4 ± 0.33
MCHC (g/dL)	38.5 ± 0.23	38.4 ± 0.57	37.7 ± 0.29	39.6 ± 1.64	42.2 ± 1.41^**$**^	37.3 ± 0.27^**+**^	38.2 ± 0.29^**+**^
PLT (10^9/L)	291 ± 63.3	326 ± 100	137 ± 42.8	96.7 ± 13.5	326 ± 137	239 ± 42.7	258 ± 49.5

Data represents Mean ± Standard Error of Mean (SEM), (*n* = 5) (One way ANOVA followed by *Tukey post* hoc test). ^*^P < 0.05 relative to Caffeine (10 mg/Kg/day); ^#^*P* < 0.05 relative to Caffeine (10 mg/Kg/day + Recovery); ^$^*P* < 0.05 relative to control; ^+^*P* < 0.05 relative to Caffeine (10 mg/Kg/day + Recovery). WBC- white blood cell, MCV- mean corpuscular volume, RBC- red blood cell, MCH- mean corpuscular hemoglobin, HGB- hemoglobin, PLT- platelets, HCT- hematocrit, MCHC- mean corpuscular hemoglobin concentration.

### Effect of caffeine on lipid profile

Table 3 shows that there were no significant differences in the serum cholesterol, triglyceride, high-density lipoprotein, and low-density lipoprotein levels during the treatment and recovery phases.

**Table 3:** Effect of caffeine on lipid profile.

Group	Cholesterol	Triglyceride	High-Density Lipoprotein	Low-Density Lipoprotein
Control	140.6 ± 10.70	62.12 ± 5.178	34.34 ± 5.919	71.13 ± 6.839
10 mg/Kg/day	102.1 ± 9.530	69.30 ± 3.161	31.86 ± 3.514	44.70 ± 4.390
20 mg/Kg/day	135.8 ± 11.81	59.12 ± 4.959	32.07 ± 4.562	69.75 ± 6.988
40 mg/Kg/day	111.2 ± 13.64	53.11 ± 2.694	28.50 ± 3.413	54.87 ± 7.665
10 mg/Kg/day + Recovery	127.9 ± 1.699	69.49 ± 2.440	25.50 ± 0.339	70.46 ± 0.936
20 mg/Kg/day + Recovery	125.6 ± 17.24	81.77 ± 22.33	29.48 ± 3.601	64.74 ± 9.849
40 mg/Kg/day + Recovery	126.8 ± 6.049	73.57 ± 11.76	27.52 ± 3.035	67.57 ± 4.707

Data expressed as Mean ± standard error of the mean (SEM) (*n* = 5 per group). ^*^P < 0.05 compared with caffeine.

### Effects of caffeine consumption on serum TNF-alpha level

**Fig. 2:**
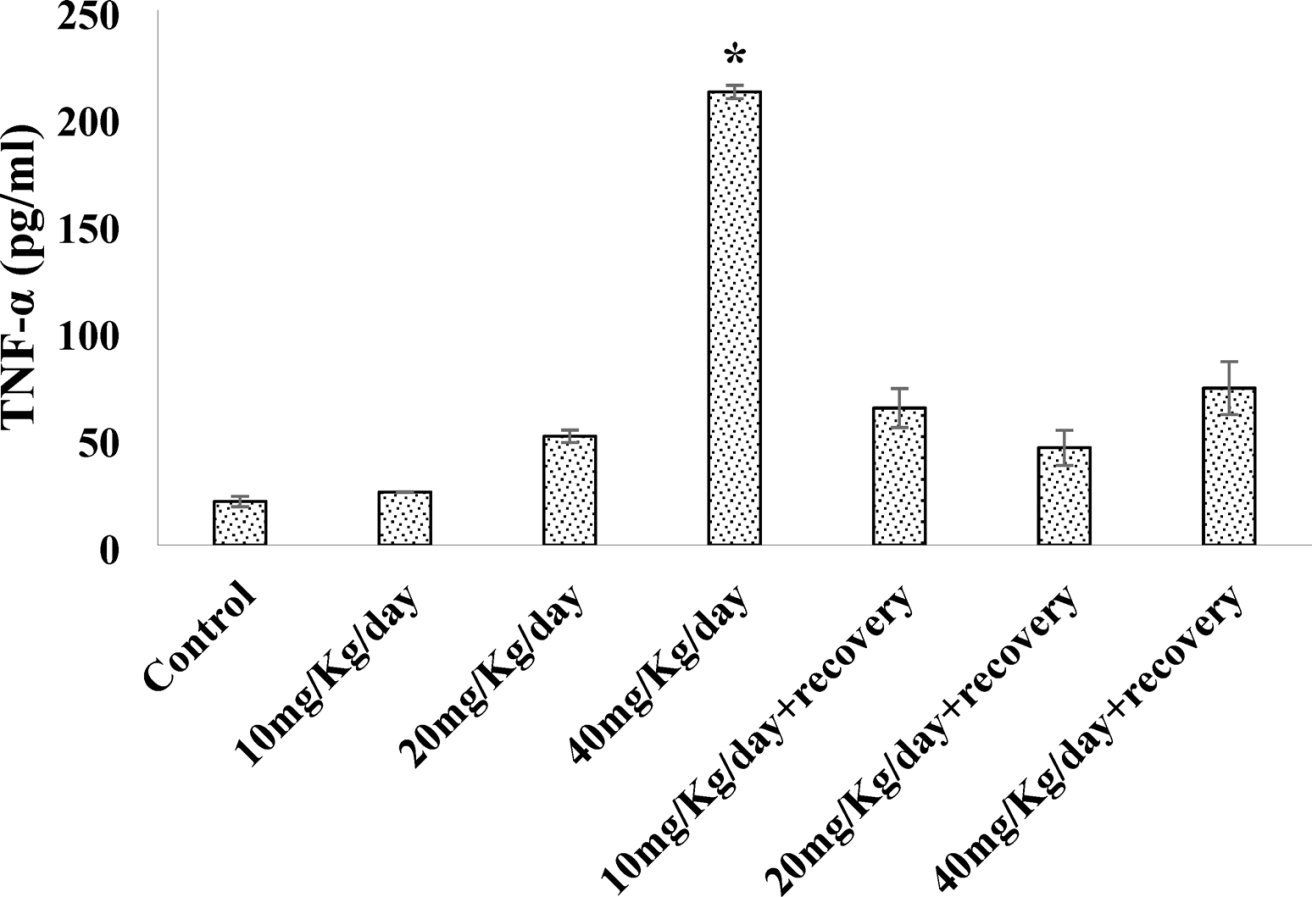
Effect of caffeine on the serum Tumor Necrosis Factor alpha (TNF-α) level. Bars expressed as Mean ± standard error of the mean (SEM) (*n* = 5 per group). The mean differences were compared by a one-way analysis of variance (ANOVA) followed by the Dunnett *post hoc test* for multiple comparisons. *P < 0.05 relative to control.

The Fig. 2 result shows a significant increase in serum TNF-alpha levels in 40 mg/kg/day caffeine treated, which reduced upon caffeine withdrawal.

### Effect of caffeine consumption on the serum levels of Gonadotropin-releasing hormone (GnRH) and Progesterone levels.

**Fig. 3:**
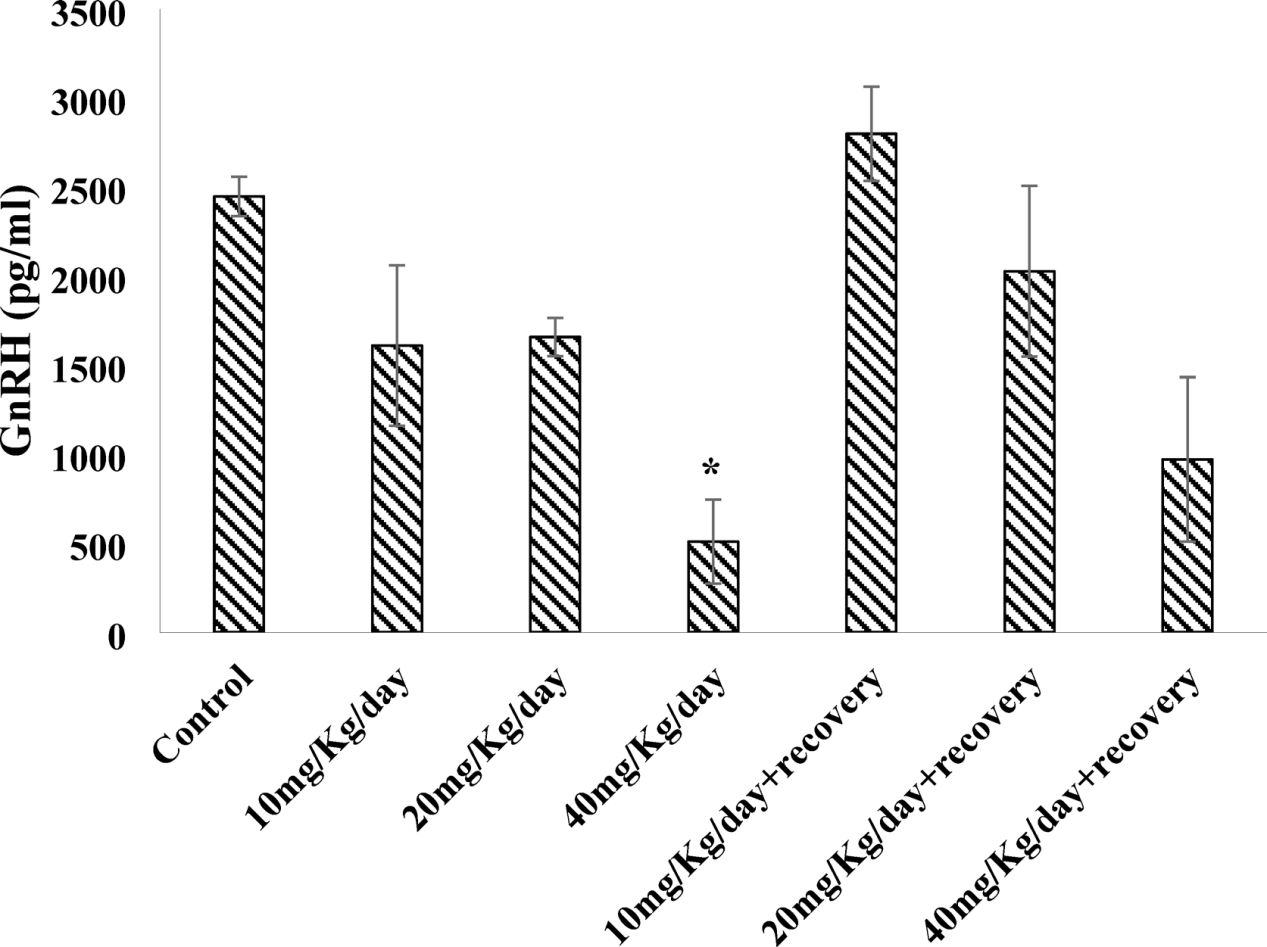
Effect of caffeine on the Serum Gonadotropin-releasing hormone (GnRH) level. Bars expressed as Mean ± standard error of the mean (SEM) (*n* = 5 per group). The mean differences were compared by a one-way analysis of variance (ANOVA) followed by the Dunnett *post hoc test* for multiple comparisons. ^*^P < 0.05 relative to control.

**Fig. 4:**
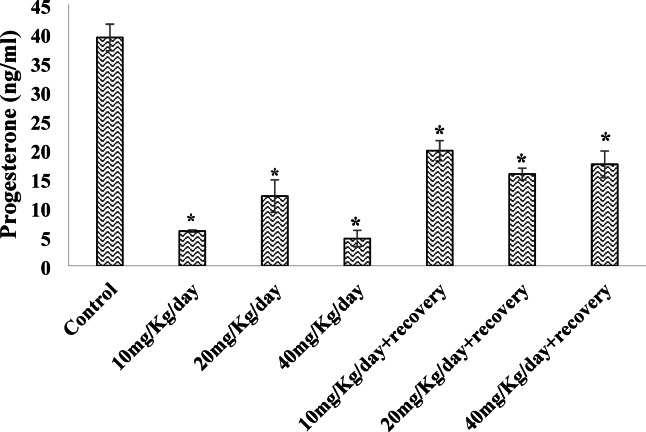
Effect of caffeine on the Serum Progesterone hormone level. Bars expressed as Mean ± standard error of the mean (SEM) (*n* = 5 per group). The mean differences were compared by a one-way analysis of variance (ANOVA) followed by the Dunnett *post hoc test* for multiple comparisons, ^*^P < 0.05 relative to control.

A significant (*P* < 0.05) decrease in serum GnRH level of rats was noted in groups treated with 40 mg/kg/day relative to the control, which was maintained after caffeine withdrawal (Fig. 3). On the other hand, treatment with caffeine significantly (*P* < 0.05) reduced progesterone levels in all treated groups compared with the control, and were still noted upon withdrawal from caffeine (Fig. 4).

### Photomicrographs of epithelial cells of the rat’s vagina

**Fig. 5:**
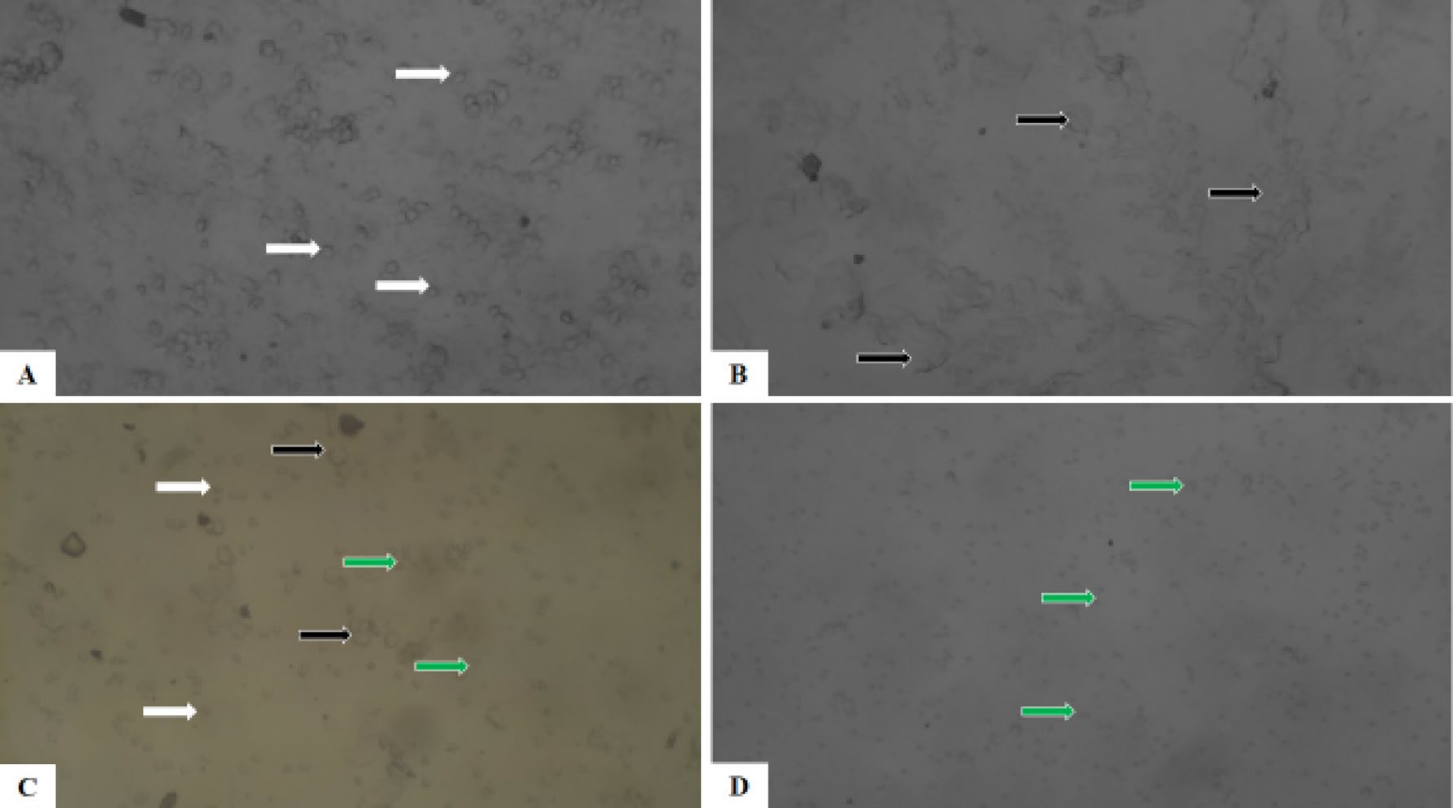
Photomicrograph of unstained vaginal smear from female rats proestrus (A), estrus (B), metestrus (C), and diestrus (D) (A) Note: Epithelial cells (white arrows), Cornified cells (black arrows), Luecocytes (green arrows). Magnification: x100.

**Fig. 6:**
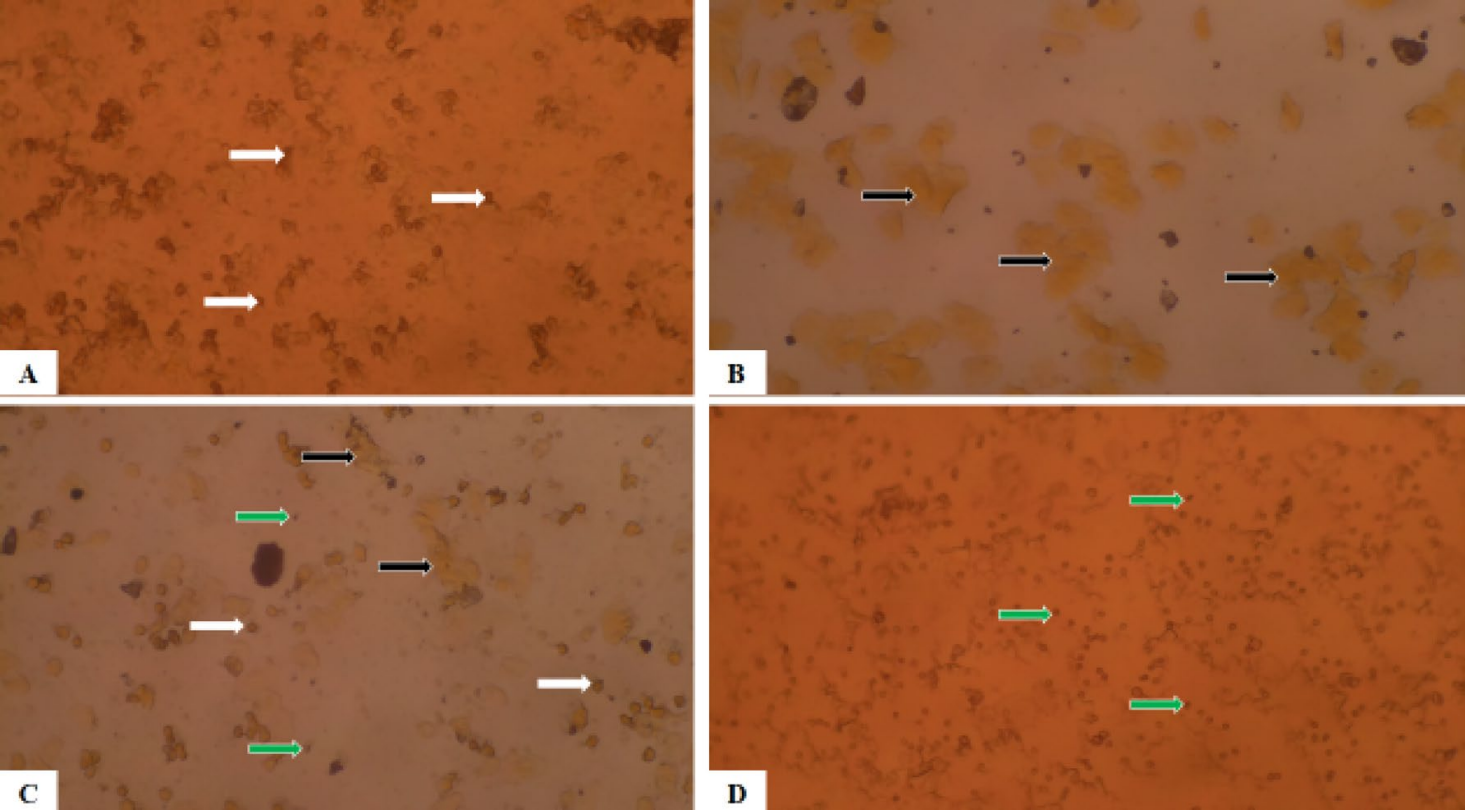
Photomicrograph of Papanicolaou’s stained vaginal smear from female rats proestrus (A), estrus (B), metestrus (C), and diestrus (D) (A) Note: Epithelial cells (white arrows), Cornified cells (black arrows), Luecocytes (greens arrows). Stained by Papanicolaou’s Magnification: x100.

The Figs. 5 and 6 are photomicrographs of unstained cells and Papanicolaou’s stained cells respectively from vaginal smears of the rats at different stages of the estrous cycle. As observed in both figures, the white arrows in the proestrus phase (A) show that nucleated epithelial cells predominated in this phase. The black arrows show anucleated cornified cells that comprise most of the estrus phase (B). The proportion of leukocytes (green arrow), cornified cells (black arrow), and nucleated epithelial cells (white arrow) is the same in the metestrus phase (C). Leukocytes comprise most of the cells during the diestrus phase (D) (green arrow).

### Effect of caffeine consumption on the frequency of occurrence of the phases of the estrous cycle

**Fig. 7:**
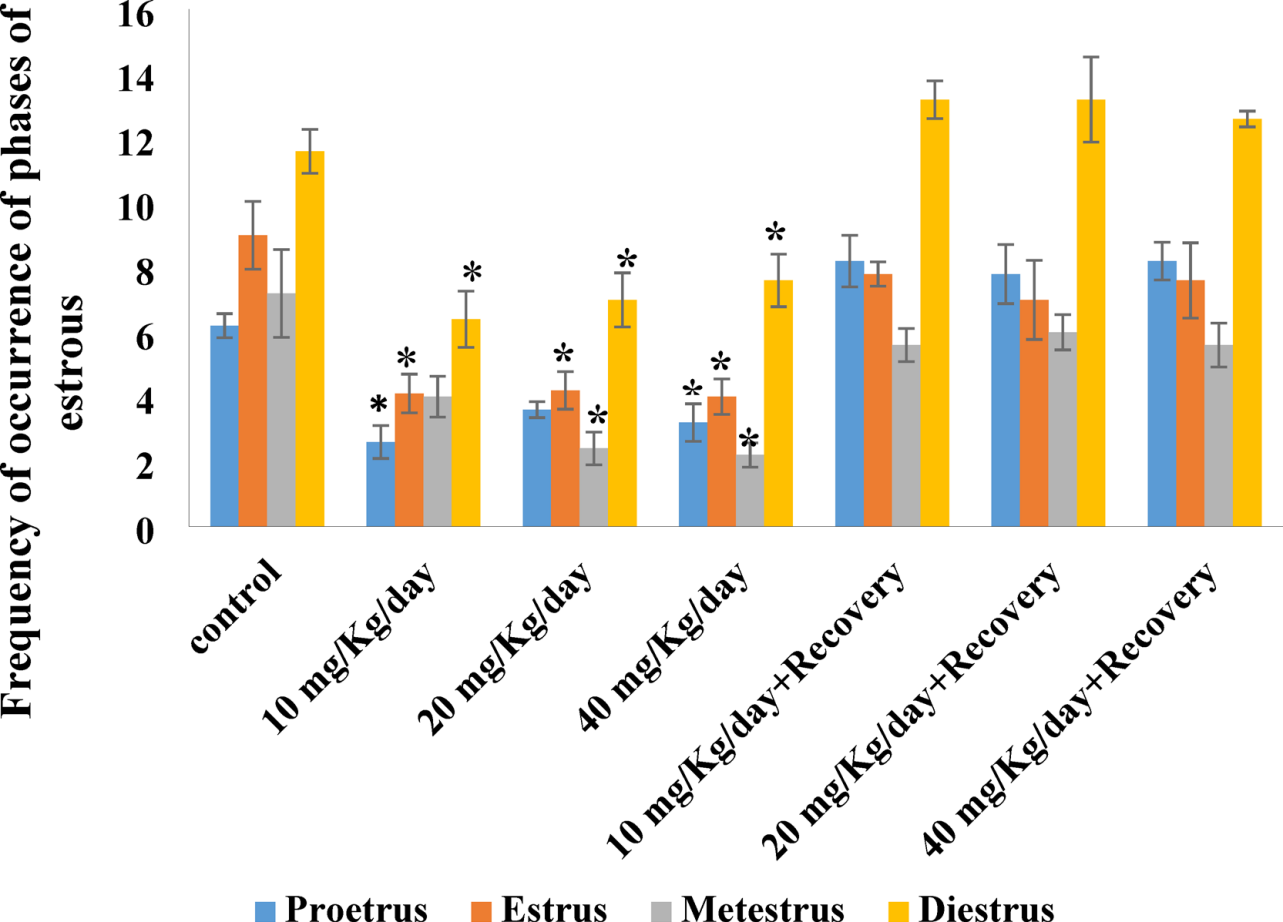
Effect of caffeine on the frequency of occurrence of phases of the estrous cycle. Bars expressed as Mean ± standard error of the mean (SEM) (*n* = 5 per group). The mean differences were compared by a one-way analysis of variance (ANOVA) followed by the Dunnett *post hoc test* for multiple comparisons. *P < 0.05 relative to control.

The result of Fig. 7 show that caffeine significantly (*P* < 0.05) reduced the frequency of occurrence of all the phases of the estrous cycle (proestrus, estrus, metestrus, and diestrus) compared with the control. Upon withdrawal of caffeine, the frequency of occurrence of these phases of estrous cycle improved.

## Discussion

The rising concerns that caffeine, a commonly consumed psychoactive substance found in many beverages, medications, and dietary supplements, is being implicated in some health issues, is the basis for this study. This study elucidates the effects of caffeine intake on the estrous cycle, hematology, and lipid profile of female Wistar rats. The findings revealed that caffeine impacted the blood, lipid profile, and some reproductive functions.

This result of this study revealed that body weight at the initial phases of caffeine administration were reduced, this reduction was persistent in the mid-dose but were reversed upon caffeine withdrawal, consistent with^37,38^ who demonstrated that caffeine had the potential of modulating metabolism and energy expenditure (thermogenesis), which led to alterations in body weight. it is plausible that the observed changes in body weights led to the reduced kidney and liver weights.

Assessment of the blood helps to understand the functions and disorders of blood and its components. This study showed alterations in white blood cell count to caffeine treatment. The decreased white blood cell counts by the highest dose contrast with the previous study of Agomuo et al.^39^., who reported that caffeine intake at higher doses caused increased WBC in a male rat. This reduced WBC may have resulted from the modulation of endothelial adhesion molecules. The insignificant reduction RBC count disagrees with an earlier study that showed caffeine levels positively correlated with increased levels of red blood cells^40^.

The insignificant reductions in the levels of cholesterol, high-density lipoprotein, and low-density lipoprotein shown in this study suggests the ability of caffeine to maintain the lipid profile level contrasting Akinola et al.^41^ who reported that triglycerides, HDL-cholesterol, and LDL-cholesterol serum levels were significantly increased in male rats treated with different percentages of caffeinated drinks after alloxan-induced diabetes. Also, Nakagawa and Pedrosa^42^ observed a significant increase in serum cholesterol after chronic caffeine treatment, but Feyisa et al.^43^. reported reduced serum triglyceride (TG) with higher coffee consumption.

Tumor necrosis factor-alpha (TNF-α) is a cytokine that plays a central role in inflammation and immune system regulation. The relationship between oxidative stress and TNF-alpha is bidirectional. TNF-alpha, aside from inducing reactive oxygen species (ROS) production, also upregulates oxidative stress, creating a feedback loop that exacerbates inflammation and cellular damage. This interaction is critical in understanding the mechanisms underlying chronic inflammatory conditions such as cardiovascular diseases, neurodegenerative disorders, and metabolic syndromes^44^. The highest dose of caffeine administered increased the TNF-α level suggesting the potential ability of caffeine to enhance oxidative stress and inflammation at high concentration and potentially implicating excess caffeine intake in disruption of normal physiological processes in line with^45^ who reported a positive association between high caffeine intake and increased inflammatory markers (TNF-α) in certain populations. On the other hand, the noted increased TNF-α level in this study was reversed during withdrawal, probably due to the restoration of adenosine receptor function, which caffeine typically antagonizes^46^. Adenosine signaling is known to suppress inflammation and oxidative stress, potentially lowering TNF-α production. Additionally, withdrawal could reduce cortisol levels and oxidative stress markers, both of which are associated with immune modulation and inflammatory cytokine regulation.

Gonadotropin-releasing hormone (GnRH) is a master regulator of reproductive function, it governs the synthesis and secretion of LH and FSH from the anterior pituitary gland and plays a central role in the regulation of the menstrual cycle, orchestrating follicular development, ovulation, and corpus luteum formation and a decrease in the levels of gonadotropin-releasing hormone disrupts the female reproductive system, as it is a vital hormone in the coordination of many reproductive functions. The reduced GnRH level revealed by the highest dose in this current study correlates with previous reports that high caffeine consumption may lead to changes in hormone secretion and menstrual irregularities^24,47^. Chronic caffeine exposure may also disrupt calcium signaling pathways critical for GnRH release, contributing to the observed effects. High doses of caffeine were found to disrupt the normal pulsatile release of GnRH, potentially leading to irregularities in menstrual cycles^48^. Though, as noted during caffeine withdrawal, the reduced GnRH level was reversed, supporting Juliano and Griffiths^49^ who reported that upon withdrawal from caffeine intake, the symptoms such as headaches, fatigue, and irritability, typically subside as well. Furthermore, GnRH and progesterone levels had been shown to gradually return to baseline after the withdrawal period^50^, highlighting the body’s resilience and its ability to restore normal hormonal balance once the stimulant is removed.

Progesterone is essential for the menstrual cycle and pregnancy; it is mainly produced by the corpus luteum in the ovary after ovulation. Progesterone plays a crucial role in preparing the endometrium for embryo implantation and supporting early pregnancy. It promotes the thickening of the endometrial lining, enhances vascularization, and suppresses uterine contractions to create a receptive environment for embryo implantation^51^. The reduced progesterone level observed with caffeine treatment and cessation of caffeine intake shows a persistent effect of caffeine on progesterone level even after withdrawal in supports of previous studies of Adelakun et al.^52^, who noted that high doses of caffeine reduced the levels of circulating progesterone in female rats via the inhibition of steroidogenesis and Purdue-Smithe et al.^53^. who reported that high caffeine intake was associated with lower luteal phase progesterone levels altering its functions, which could unfavorably impact fertility and menstrual cycle regularity. Also, it is plausible that caffeine interacted with the hypothalamo-pituitary-adrenocortical axis, activating it and increasing cortisol levels, which probably inhibited steroidogenesis and reduced progesterone synthesis. Elevated cortisol disrupts hormonal feedback loops, further impacting reproductive hormones^54^. Aside from the dosage of caffeine administered, other factors such as the duration of the experiment, timing of hormone measurement^55^, interaction with other nutrients and diet^56^, stress, and experimental conditions^57^ are possible factors that may alter the levels of progesterone. The potential of caffeine to adversely alter progesterone levels irreversibly suggests that females should apply some caution in consuming caffeine for the essential role that progesterone plays in the menstrual cycle.

Vaginal smears are useful for precisely identifying the types of cells present in each stage of the estrous cycle when combined with various staining techniques like Hematoxylin and Eosin (H&E) stain, Papanicolaou (PAP) stain, Giemsa, and toluidine blue^33^. In comparison with Marcondes principle, vaginal smear staining allows researchers to see and distinguish between mast cells, nucleated epithelial cells, cornified cells, and leokocytes at various stages of the estrous cycle in female Wistar rats using better resolution with well-defined features. The four phases of the estrous cycle appeared normal in the two methods that were used for assessment, but the observations were clearer in the stained cells. The proestrus phase was characterized by small, rounded, nucleated epithelial cells of uniform appearance. The majority of the big cornified cells in the estrus phase were non-nucleated, appearing as uneven sheets and clusters in vast numbers. Three different cell types were visible during the metestrus phase: leukocytes, cornified cells, and nucleated epithelial cells. Usually in contact with other cell types, leucocytes form tiny, densely packed clumps of cells. According to Ajayi et al.^57^, the leukocytes were the primary cell type seen throughout the diestrus phase. The reported decreases in the frequency of occurrence of all phases of the estrous cycle in this study support findings of previous studies that caffeine consumption could alter the frequency of occurrence of the various phases of the estrous cycle^58,59^. These decreases were probably due to the effect of caffeine on hormonal patterns. Previous researches indicate that consuming large amounts of caffeine may alter hormone release and cause irregular menstruation^24,58,60^. Remarkably, during caffeine withdrawal, the frequency of occurrence of all the phases of the estrous cycle was reversed (increased), suggesting that caffeine consumption did not have a permanent effect on the estrous cycle.

### Limitations of the study

This study employed the use of a smaller number of animals per group, and it was impossible to verify all phases of the estrous cycle during the analysis time of each vaginal smear at the time this study was conducted. Further studies will take these into consideration for a more comprehensive result.

## Conclusion

The findings of this study show that caffeine exerted adverse alterations on the estrous cycle, reduced WBC count while maintaining the lipid profile. These alterations were however reversed upon withdrawal from caffeine, hence, this study highlights the facts that given the potential implications for metabolic health, immune function, cardiovascular, and reproductive risk, the stoppage of caffeine intake by a female can result in restoration of the altered changes overtime.

## Data Availability

The datasets used and/or analysed during the current study available from the corresponding author on reasonable request.
